# Brain metastases of sarcoma: a rare phenomenon in rare tumours

**DOI:** 10.1007/s00432-023-05451-1

**Published:** 2023-11-23

**Authors:** Wiktoria Jędrys, Aleksandra Leśniak, Aneta Borkowska, Piotr Rutkowski, Paweł Sobczuk

**Affiliations:** 1 Department of Soft Tissue/Bone Sarcoma and Melanoma, Maria Skłodowska-Curie National Research Institute of Oncology in Warsaw, Warsaw, Poland; 2 Faculty of Medicine, Medical University of Warsaw, Warsaw, Poland

**Keywords:** Brain metastases, Sarcoma, Surgery, Radiotherapy

## Abstract

The usual site for distant metastases of sarcoma is lungs, while brain metastasis (BM) occurs much less frequently and usually late in the disease progression. Despite the advancement in cancer treatment, the outcome for patients with brain metastasis is poor, and their lifespan is short. The frequency of BM in sarcoma seems to be affected by the location and histology of the primary tumour. Sarcoma subtypes with a high propensity for brain metastasis are ASPS, leiomyosarcoma and osteosarcoma. There are no clear guidelines for the treatment of sarcoma brain metastasis. However, therapeutic options include surgery, radiotherapy and chemotherapy, and are often combined. Targeted therapies are a promising treatment option for sarcoma but require investigation in patients with BM. The following review presents the data on sarcoma brain metastasis incidence, treatment and prognosis.

## Introduction

Sarcomas are rare tumours with an annual incidence in Europe of 4–5/100 000 (Gatta et al. 2017). Recent decades have brought better diagnostic possibilities, allowing for early detection, more accurate staging, and new treatments that increase curability and prolong survival. Sarcomas are very heterogeneous group, with over 100 different subtypes distinguished in the recent WHO classification, including leiomyosarcoma, liposarcoma and undifferentiated pleomorphic sarcoma as the most common. Approximately, 30–50% of patients with localised sarcoma will eventually relapse or develop distant metastases. The median overall survival of patients with metastatic disease is around 2 years, but can vary depending on histologic subtype (Lavit et al. 2022; Lin et al. 2020; Lochner et al. 2020; Sobczuk et al. 2020).

With the advancements in therapies, uncommon brain metastases are detected more often (Siontis et al. 2019). It is estimated that about 1–8% of sarcoma patients can develop brain metastases in the course of the disease, and most of them will die within 12 months (Salvati et al. 2010; Shweikeh et al. 2014). To date, no clear guidelines for the treatment of brain metastases from sarcoma have been established. This study aims to provide an update on the current state of art on epidemiology, diagnosis and treatment of sarcoma brain metastases.

## Epidemiology and biology of brain metastases in sarcoma

The incidence of brain metastases (BM) in cancer patients is 10–30% making it a relatively common cancer complication (Siegel et al. 2014). However, BM from sarcoma occurs much less often, with an incidence of about 1–8% (Nakamura et al. 2011; Salvati et al. 2010). The usual site for sarcoma distant metastases are the lungs, followed by the liver and bone. Lung metastases precede BM in about 51–80% of cases (Ogose et al. 1999; Salvati et al. 2010; Yoshida et al. 2000).

The location and histology of the primary tumour seem to affect the frequency of BM in sarcoma. According to a study by Yoshida et al., BM were found in 7.2% of patients with soft tissue sarcomas (STS) but only in 3.5% of patients with bone sarcomas (BS) (Yoshida et al. 2000). However, in the paediatric population, where bone sarcomas are more common, the incidence of BM was 2.28% for STS and 5.9% for bone sarcomas (Postovsky et al. 2003). In smaller studies concerning single sarcoma subtypes, the incidence occasionally exceeds the abovementioned percentages. Additionally, the proportions differ significantly between different subtypes, i.e., for alveolar soft part sarcoma (ASPS), the incidence of BM is between 16 and 30% and for Ewing's sarcoma, 3.3–40% (Al Sannaa et al. 2016; Chou et al. 2011; Malouf et al. 2019; Mendes et al. 1999; Parasuraman et al. 1999; Paulino et al. 2003; Yoshida et al. 2000).

Sarcomas are a heterogeneous group of more than 50 different histological types (von Mehren et al. 2020). The most commonly occurring ones in the adult population are liposarcoma, leiomyosarcoma, and undifferentiated pleomorphic sarcoma (Siegel et al. 2014). In adolescents and children, osteosarcoma is the most common malignant bone tumour, while rhabdomyosarcoma comprises more than 50% of paediatric soft tissue sarcomas (Park et al. 2008; Taran et al. 2017). The most common subtypes differ between the reports regarding the propensity to metastasize to the brain, depending on which subtypes were included in the analyses. In Gusho et al. study, where 5933 sarcoma patients were analyzed, 44 (7.4%) developed BM. Unspecified sarcoma (18.2%), leiomyosarcoma (15.9%) and hemangiosarcoma (15.9%) were reported to metastasize to the brain most often (Gusho et al. 2021). In a different study where 112 sarcoma cases with BM were analyzed, the most common subtypes were undifferentiated sarcoma (27%), ASPS (16%), and osteosarcoma (13%) (Al Sannaa et al. 2016) (see Table 1).

**Table 1:** Sarcoma subtypes most frequently mentioned in terms of BM, percentage of patients developing BM, percentage of all sarcoma patients with BM

Sarcoma subtype	Percentage of patients developing BM from sarcoma subtype (%)	Percentage of all sarcoma patients with BM (%)	References
Liposarcoma	1.2–15%	2–12.5%	Al Sannaa et al. (2016), Bailey et al. (2001), Chaigneau et al. (2018), Chou et al. (2011), Espat et al. (2002), Salvati et al. (2010), Yoshida et al. (2000)
Leiomyosarcoma	2–20%	7–20%	Al Sannaa et al. (2016), Chaigneau et al. (2018), Chou et al. (2011), Espat et al. (2002), Gonzalez et al. (2021), Gusho et al. (2021), Salvati et al. (2010), Tigchelaar et al. (2022), Tirumani et al. (2014)
Alveolar soft part sarcoma (ASPS)	16–43.4%	8.5–18.2%	Al Sannaa et al. (2016), Chou et al. (2011), Fox et al. (2009), Gonzalez et al. (2021), Malouf et al. (2019), Postovsky et al. (2003), Salvati et al. (2010), Sood et al. (2014)
Malignant peripheral nerve sheath tumours (MPNST)	0.5–6.67%	3–4.6%	Acem et al. (2021), Al Sannaa et al. (2016), Chaigneau et al. (2018), Chou et al. (2011), Gonzalez et al. (2021), Puffer et al. (2016), Xu et al. (2021), Yoshida et al. (2000)
Angiosarcoma	ND	4–13.6%	Al Sannaa et al. (2016), Chaigneau et al. (2018), Gonzalez et al. (2021)
Undifferentiated pleomorphic sarcoma	2.04–10%	4.6–11%	Al Sannaa et al. (2016), Chou et al. (2011), Espat et al. (2002), Gonzalez et al. (2021), Salvati et al. (2010)
Osteosarcoma	1.9–6.5	13–13.6%	Al Sannaa et al. (2016), Bekiesinska-Figatowska et al. (2017), Chou et al. (2011), Gusho et al. (2021), Paulino et al. (2003), Yonemoto et al. (2003), Yoshida et al. (2000)
Ewing's sarcoma (ES)	1–40%	8–14%	Al Sannaa et al. (2016), Bekiesinska-Figatowska et al. (2017), Chou et al. (2011), Malouf et al. (2019), Mendes et al. (1999), Paulino et al. (2003), Salvati et al. (2010), Yoshida et al. (2000)
Rhabdomyosarcoma	2.4–18%	4–10%	Al Sannaa et al. (2016), Baram et al. (1988), Bekiesinska-Figatowska et al. (2017), Espat et al. (2002), Parasuraman et al. (1999), Paulino et al. (2003), Salvati et al. (2010), Yoshida et al. (2000)
Chondrosarcoma	0–2%	3%	Al Sannaa et al. (2016), Bekiesinska-Figatowska et al. (2017), Chou et al. (2011), Ogose et al. (1999), Yoshida et al. (2000)

Sarcomas can metastasize to the brain through the hematogenous spread, affecting brain parenchyma and possibly forming multiple lesions, or through the direct extension of the tumour from metastases located in skull bones or the meninges ("Erratum: De B, Kinnaman MD, Wexler LH, Kramer K, Wolden SL. Central nervous system relapse of rhabdomyosarcoma. Pediatr Blood Cancer. 2017; https://doi.org/10.1002/pbc.26710," 2017; Kebudi et al. 2005; Postovsky et al. 2003). A study of patients with Ewing's sarcoma found that nine out of 13 patients with autopsy-confirmed CNS involvement had evidence of the direct expansion of tumour from bone metastases (Kies and Kennedy 1978). Parenchymal lesions were only present in three patients. Consistently, in another study, 21 out of 23 rhabdomyosarcoma patients with CNS relapse had evidence of leptomeningeal spread, while 2 had only parenchymal involvement ("Erratum: De B, Kinnaman MD, Wexler LH, Kramer K, Wolden SL. Central nervous system relapse of rhabdomyosarcoma. Pediatr Blood Cancer. 2017; https://doi.org/10.1002/pbc.26710," 2017). In paediatric patients with rhabdomyosarcoma and Ewing's sarcoma, a direct extension of the tumour from the skull and meninges was found in half of the patients. In contrast, the other half had evidence of haematogenous spread (Parasuraman et al. 1999).

There are no clear molecular reasons why CNS metastases of sarcoma are rare (Chua et al. 2016). When they do occur, brain metastases coexist with prior metastases to other sites, with 80–97.1% of patients already having extracranial metastatic disease (Kokkali et al. 2021; Ogose et al. 1999; Patrikidou et al. 2020; Yoshida et al. 2000). Given that brain metastases are usually preceded by pulmonary metastases, it has been proposed that the hematogenous spread of sarcoma can explain this association (Kebudi et al. 2005).

It has been suggested that an increased incidence of sarcoma brain metastases may result from successful chemotherapy, resulting in longer overall survival of patients with sarcoma (España et al. 1980). However, a study of 254 children with osteosarcoma treated between 1962 and 1989 did not find a statistically significant increase in the incidence of CNS metastases after the introduction of multi-agent therapy in 1982 (Marina et al. 1993).

A study of 3829 patients with sarcoma demonstrated that extremity and trunk soft-tissue sarcomas have a propensity for distant metastases (notably pulmonary), while the spread of visceral and retroperitoneal sarcomas tends to manifest as local recurrences (Espat et al. 2002). This is supported by observations from another study that sarcomas located in the extremities were the most frequent origin of brain metastases, found in 50% of the cases, followed by visceral and retroperitoneal (27%), trunk (12%), and head and neck (11%) (Al Sannaa et al. 2016).

The distribution of brain metastases of sarcoma follows general patterns of brain metastases, where 80% is found in the cerebral hemispheres, 15% in the cerebellum, and 5% in the brainstem. This reflects the blood distribution in the CNS (Subramanian et al. 2002). The majority of brain metastasis in sarcoma patients is located in the cerebrum. A cohort study from the Hellenic Group of Sarcomas and Rare Cancers found that 58.8% of patients with sarcoma metastasizing to the brain had supratentorial lesions (Kokkali et al. 2021). In a study by Salvati et al., the frontal lobe was the most frequent location of the lesions, comprising 68.5% of the cases, followed by the parietal (17.1%), occipital (8.5%) and temporal (8.5%) lobes (Salvati et al. 2010).

Cerebellar metastases of sarcoma are rare. Subtentorial lesions were only present in 16.6% of children with sarcoma involving the CNS (Postovsky et al. 2003). This aligns with another study, where cerebellar metastases developed in 2 out of 14 patients with sarcoma (Chan et al. 2020). Interestingly, these patients were the only patients in this study to survive more than 8 months after brain metastasis diagnosis.

Sarcoma metastases in the brainstem are highly uncommon, and only one such case has been reported in a 59-year-old patient with uterine leiomyosarcoma (Mawrin et al. 2002). However, the incidence rate of brainstem lesions can be underestimated.

In most cases, metastatic lesions are solitary. Single brain metastasis has been reported in 38.2–70.3% of patients (Chou et al. 2011; Kokkali et al. 2021; Ogose et al. 1999; Postovsky et al. 2003; Yoshida et al. 2000). A more detailed examination of the distribution of brain metastases from the Hellenic Group of Sarcomas and Rare Cancers cohort study found a single lesion in 38.2%, 2–4 lesions in 32.4%, and more than four metastases in 20.6% of patients (Kokkali et al. 2021). Notably, a group developing a modified, sarcoma-specific GPA index determined that the number of CNS metastases (1, 2–4, or more than 4) significantly affected the median overall survival, where the smaller number of lesions indicated a better prognosis (Patrikidou et al. 2020).

## Diagnostics and screening

Diagnosis of brain metastases is usually based on neuroimaging, mainly magnetic resonance imaging (MRI) or computed tomography (CT) (Gronchi et al. 2021; von Mehren et al. 2018). Most patients (77–100%) display neurological symptoms before BM diagnosis (Chua et al. 2016; De et al. 2018; Marina et al. 1993). Most exhibit more than one symptom (Chua et al. 2016). In a study of 114 sarcoma patients, all 11 patients diagnosed with brain metastases had initial neurological symptoms. No brain lesions were detected at autopsy in 40 patients without clinical signs of brain metastases (España et al. 1980).

However, the incidence of asymptomatic brain metastases may be higher than reported, as brain imaging is typically only performed if neurological symptoms are present, indicating the possibility of CNS involvement (Postovsky et al. 2003). In 35 cases, 12% of patients had no neurological symptoms, and CNS metastases were only detected by radiological screening (Salvati et al. 2010). On the other hand, neurological symptoms or even abnormalities in brain imaging studies do not always indicate the presence of brain metastases. In a study of patients with metastatic rhabdomyosarcoma, 7 of 56 (12.5%) patients that underwent brain imaging at diagnosis had abnormal scans. Four of those seven patients had symptoms suggesting metastases in the head or neck. Parenchymal brain metastases were only confirmed in one patient (Spunt et al. 2001).

Most neurological symptoms patients demonstrate are non-specific (De et al. 2018). The most common symptom is a headache, present in 39–52.9% of patients (Flannery et al. 2010; Kokkali et al. 2021; Mehta and Hendrickson 1974; Salvati et al. 2010). Chua et al. found that the most frequent symptoms that patients presented with were headache (43%), seizures (29%), dizziness (29%), nausea and vomiting (21%), focal weakness (21%), and change in mental status (Chua et al. 2016). Other reported symptoms included paraesthesias, back pain, double vision, and blurred vision (Mehta and Hendrickson 1974).

Neurological examination may also reveal signs of cerebral spread in patients with sarcoma. Mehta et al. noted that papilledema was the most common symptom observed in patients with Ewing's sarcoma metastatic to the brain, followed by paraparesis, hemiparesis, and vertebral column tenderness (Mehta and Hendrickson 1974). Salvati et al. reported that motor deficits were found in 86% of patients and were the most frequent neurological signs (Salvati et al. 2010).

Neurological symptoms are more pronounced in children. In a study of brain metastases in the paediatric population, neurological manifestations were more severe than those reported in adult patients, with 60% of children experiencing seizures (Kebudi et al. 2005). The onset of neurological symptoms was also more sudden. It has been suggested that more rapid kinetics of tumour growth in childhood neoplasms may explain such differences (Bouffet et al. 1997).

Given the rarity of sarcoma brain metastasis, current guidelines do not recommend routine brain imaging for all sarcoma patients without neurological signs or symptoms that indicate possible CNS involvement (Bailey et al. 2001). Moreover, brain metastases usually occur after the systemic spread of sarcoma at the advanced stages of the disease. In a study by Yoshida et al., only 18.5% of sarcoma patients with brain metastases died due to CNS progression (Bindal et al. 1994). The remaining patients died from systemic progression (70.4%) or infections (11.1%). In another study, brain metastases were confirmed at autopsy as a cause of death in 4 out of 11 patients (Parasuraman et al. 1999). Therefore, it has been argued that neuroimaging should only be performed when neurological symptoms are present because early diagnosis of brain metastases does not increase overall survival [34, 40].

However, the high incidence of brain involvement in specific histological subtypes of sarcoma, mainly ASPS, prompted the researchers to suggest that screening for brain metastases should be performed in all patients with those sarcomas (Hoiczyk et al. 2014). Others propose that screening for brain metastases could be utilized in patients with systemic spread of osteosarcoma or malignant fibrous histiocytoma (Shido and Matsuyama 2017; Shweikeh et al. 2004). Yonemoto et al. (2003) and Marina et al. (1993) suggest that osteosarcoma patients with active pulmonary metastases should undergo routine neuroimaging.

According to NCCN Guidelines for Soft Tissue Sarcoma guidelines, CNS MRI or CT should be performed in patients with ASPS, mainly if pulmonary metastases exist [53]. According to ESMO-EURACAN-GENTURIS Clinical Practice Guidelines, it can also be considered for patients with clear-cell sarcoma and angiosarcoma (Schoffski et al. 2018).

## Treatment

Standard treatment of localized sarcomas is based on surgery, radiotherapy, and sometimes chemotherapy. Multimodal therapies are implemented depending on the feasibility of resection, sarcoma advancement, and the patient's health status. In the case of oligometastatic disease, the goal is to resect solitary metastases, optionally followed by chemotherapy. Still, in terms of multiple metastases, the preferred therapy is systemic therapy, mainly chemotherapy-based (Gronchi et al. 2021). Unluckily brain metastases are characterized by high radio- and chemoresistance, making the therapy more challenging (Fox et al. 2009). While multimodal therapies for sarcoma result in prolonged survival, the prevalence of brain metastasis increases due to poor CNS penetration of systemic agents (Siontis et al. 2019). There is no standard treatment recommendation for patients with sarcoma brain metastases, but an individualized approach is recommended depending on the sarcoma subtype.

### Surgery

In resectable metastasis, surgery is the primary intervention. Median survival without surgical resection is 1.2–2.7 months, while after metastasectomy, it increases, ranging from 5.4 to 25.8 months (Chou et al. 2011; Espat et al. 2002; Salvati et al. 2010; Yoshida et al. 2000). Only one study of 14 patients showed no significant difference in survival between patients who did and did not undergo surgery (Chan et al. 2020).

Individual anatomy, location of BM, and the patient's clinical condition may affect the possibility of complete resection and, as a result, patient survival. According to Bindal et al., a preoperative Karnofsky Performance Score (KPS) of < 70 resulted in 6.6 months of survival, while a KPS of > 70 resulted in 15.7 months. Moreover, patients who underwent complete resection survived 14.0 versus 6.2 months for incomplete resection (Bindal et al. 1994). On the contrary, according to Fox et al., there is no statistical difference in survival between patients who receive total resection and those where only specific lesions were targeted (Fox et al. 2009). A few studies showed that complete resection was possible in 85–95% of patients undergoing surgery, suggesting BM from sarcoma may have characteristics facilitating its removal (Deguchi et al. 2020; Fox et al. 2009; Salvati et al. 2010).

Likely, evidence of lung metastases at the time of craniotomy is not a contradiction for surgery and does not significantly change patients' survival. The mean survival for patients with craniotomy and concurrent lung metastases was 8.6–11.8 vs 10.4–10.5 for diseases limited to the brain (Bindal et al. 1994; Salvati et al. 1998).

Apart from improving survival, metastasectomy has been reported to reduce neurological symptoms in most patients (Baram et al. 1988; Ogose et al. 1999; Yoshida et al. 2000). Also, according to Deguchi's study, craniotomy improved the KPS in 50% of cases, especially those with low preoperative performance (Deguchi et al. 2020).

### Radiotherapy

Sarcoma metastasizing to the central nervous system is considered to be relatively radioresistant (Ahmad et al. 2016; Chou et al. 2011). Nevertheless, radiotherapy has been widely used in treating sarcoma BM, both therapeutically and palliatively.

Two main methods of radiotherapy are whole-brain radiotherapy (WBRT) and stereotactic radiation therapy (SRT) in one fraction (radiosurgery) or more than one fraction (hypofractionated stereotactic radiation therapy). Whole-brain radiation therapy uses low doses of radiation per fraction to indiscriminately irradiate whole brain tissue, typically used in cases with multiple intracranial malignancies (Kumar et al. 2018). In contrast, SRT is a precise technique that utilizes focused radiation to target a limited quantity of small lesions, enabling a higher biologically effective dose (BED) on the lesion. WBRT is linked to neurological adverse effects, such as fatigue, cognitive impairment, and memory loss (Kumar et al. 2018). In comparison, the localized impact area of SRT has the benefit of decreased side effects, despite utilizing higher doses of radiation (Kumar et al. 2018). However, the use of SRT is limited by the number and size of targets, as treating a higher volume of disease may increase the risk of radiation necrosis (Kumar et al. 2018). Although SRT is usually recommended for patients with 1–4 lesions, it has been considered an option in patients with up to 10 brain metastases (Yamamoto et al. 2014) as long as the total volume of the disease remains low (Kumar et al. 2018). As a result, patients with a small number of brain metastases can be treated with SRT, while WBRT is mainly used in patients with disseminated cerebral metastases (Farooqi et al. 2020). It is important to remember that SRT does not exclude future WBRT, and vice versa: WBRT does not preclude later SRT. In selected cases, it is also possible to re-treat the previously irradiated lesion.

#### Stereotactic radiation therapy (SRT)

Sim et al. reported that SRT treatment of brain metastases from sarcoma resulted in good local control, 89% after 1 year (Sim et al. 2020). However, distant control (34%) and overall survival (38%) remained poor. Flannery et al. found that patients treated with Gamma Knife radiosurgery had 88% local control for a median interval of 4 months, with 61% of 1-year OS (Flannery et al. 2010). It has also been noted that lesions showing evidence of regression after SRT tended to be initially smaller than stable or progressive tumours (mean volume 3 cm^3^ vs 11.2 cm^3^).

In 2018 Xia et al. explored the ability of local radiation and anti-PD-1 blockade to induce an anti-tumour response in a preclinical study on mice with osteosarcoma brain metastases. The combination treatment produced a more robust systemic anti-tumour response than either treatment alone and created the prospect of new treatment options (Xia et al. 2018). However, the results apply only to an animal model, and so far, human clinical trials with anti-PD-1 pembrolizumab in osteosarcoma showed no clinically significant antitumor activity (Boye et al. 2021; Tawbi et al. 2017).

#### Whole-brain radiotherapy (WBRT)

Whole-brain radiotherapy can be used in cases where surgery and SRT are unsuitable or as a part of multi-modality treatment. In the cohort study from the Hellenic Group of Sarcomas and Rare Cancers, which evaluated the impact of different treatment modalities, whole-brain radiation therapy was associated with longer overall survival than no WBRT (Kokkali et al. 2021).

Since surgery and SRT are local treatments, their impact on the areas outside the treatment field remains limited. Therefore, combining these modalities with WBRT may result in better outcomes. The addition of WBRT to SRT improved intracranial control from 50 to 85% at 1 year (Farooqi et al. 2020). On the other hand, another study found no statistical difference in the overall survival of patients who did and did not receive WBRT after surgery (Wroński et al. 1995).

Even if radiation therapy does not significantly prolong survival, it may be used as palliative treatment. Symptoms were alleviated in six out of 7 patients with Ewing's sarcoma who received palliative radiotherapy and remained symptom-free until death (Mehta and Hendrickson 1974). In another study, two out of 5 patients who underwent radiotherapy showed marked improvement in neurological symptoms and died of systemic disease (Ogose et al. 1999).

### Systemic therapy

Late presentation of brain metastases during sarcoma progression and the presence of disseminated disease at the time of BM diagnosis underscores the need for systemic treatments. In a study by Yoshida et al., most sarcoma patients (70.4%) died from systemic progression, with only 18.5% due to direct CNS involvement (Bindal et al. 1994). Chemotherapy is, therefore, widely used in treating sarcoma, including its distant metastases, but its effectiveness remains limited. Doxorubicin, ifosfamide, gemcitabine, and docetaxel are commonly used in patients with sarcoma (Chaigneau et al. 2018; Ratan and Patel 2016). They cross the blood–brain barrier poorly, restricting their usefulness in treating brain metastases (Ahmad et al. 2016). Furthermore, CNS involvement is usually the final stage in the progression of the disease (Kokkali et al. 2021) and diagnosis of brain metastases is preceded by a median number of two lines of systemic therapies (Kokkali et al. 2021). Therefore, most patients had already been treated with systemic treatment by the time of brain metastasis, which further affects the efficacy of this line of therapy (Siontis et al. 2019).

Histological subtypes impact the tumour's sensitivity to chemotherapy, resulting in some types (e.g. osteosarcoma and Ewing's sarcoma) being more chemosensitive while others (e.g. clear-cell sarcoma) remain chemoresistant (Chaigneau et al. 2018). A study of aggressive multidisciplinary treatments for patients with osteosarcoma metastasizing to the brain found that chemotherapy is a favourable prognostic factor for longer overall survival, independently of surgery and radiotherapy (Zhu et al. 2021). This is in line with results from a multicentre study of 246 sarcoma patients with CNS metastases, which also demonstrated a positive impact of chemotherapy on overall survival (Chaigneau et al. 2018). However, there was no evidence of chemotherapy influencing survival in a study reviewing the data from the Surveillance, Epidemiology, and End Results (SEER) database (Gusho et al. 2021).

There is some evidence of survival benefits for patients whose locally aggressive treatment was supplemented with chemotherapy. In a study of 112 patients, combining chemotherapy with surgery improved overall survival significantly, compared with combining radiotherapy with surgery or surgery alone (Al Sannaa et al. 2016). This was partially confirmed by Zhu et al. (2021), who observed the advantage over solely performed metastasectomy but failed to show the difference compared to surgery with adjuvant radiotherapy.

In recent years, targeted therapies, including sunitinib and pazopanib, have emerged as a promising treatment option for soft-tissue sarcomas (Nathenson et al. 2018; Ratan and Patel 2016; van der Graaf et al. 2012). ASPS has been shown to be sensitive to tyrosine kinase inhibitors, such as sunitinib or crizotinib (Roulleaux Dugage et al. 2021; Schoffski et al. 2018). Another study investigating the effects of combined axitinib (antiangiogenic tyrosine kinase inhibitor) and pembrolizumab (anti-PD1 immune checkpoint inhibitor) treatment in patients with sarcoma found promising activity in ASPS patients (Wilky et al. 2019). However, an analysis of the French Sarcoma Group showed that among ASPS patients treated with antiangiogenic therapies (sunitinib, pazopanib, and cediranib), those with brain metastases had significantly shorter progression-free survival than those without BM, suggesting that patients with ASPS developing brain metastases have limited benefit from this form of treatment (Malouf et al. 2019). A case report regarding a 37-year-old patient with ASPS treated with pazopanib also reported the detection of brain metastases after 35 months of treatment, despite achieving a response in the tumour and lung metastases (Shido and Matsuyama 2017). Further studies are needed to investigate the possible benefits of targeted therapies in treating sarcoma metastatic to the brain. Currently, no dedicated studies are investigating targeted drug therapies for sarcomas metastasizing to the central nervous system (Zhu et al. 2021).

Moreover, there is some evidence for the activity of immunotherapy in selected subgroups of patients. A meta-analysis of 27 studies showed the potential benefits of immune checkpoint inhibitor therapy, with ASPS, UPS and Kaposi sarcoma being the most susceptible (Saerens et al. 2021). A study of 80 patients with soft-tissue and bone sarcomas treated with pembrolizumab (anti-PD1 antibody) reported a response in 40% of patients with UPS, compared with 18% of all patients with STS and 5% of patients with bone sarcoma (Tawbi et al. 2017). However, the effects of these therapies in patients with BM have not yet been investigated. Additionally, patients with symptomatic BM are usually excluded from clinical trials.

## Outcomes and prognostic factors

The 5-year survival rate in localized STS and bone sarcoma reaches 75–90% and 80%, respectively. However, in the case of distant metastases, it decreases markedly to 15–20% in STS and 20–60% in bone sarcoma (Howlader et al. N, based on November 2019 SEER data submission, posted to the SEER web site, April 2020). The median overall survival for advanced STS is 8–15 months (Hoiczyk et al. 2014; Italiano et al. 2011; Meyer and Seetharam 2019). Brain metastases are usually the last stage in the disease progression, and the outcome for the patients is poor (Salvati et al. 2010). With only the best supportive care, the median survival is between 1.2 and 2.7 months (Chua et al. 2016; Espat et al. 2002).

Standard prognostic factors, i.e. the GPA index, were established to guide the treatment of brain metastases from primary tumours such as lung or breast cancer. They were developed to be used across multiple histologies and molecular tumour subtypes. According to Patrikidou's study, the original GPA index is not prognostic for sarcoma BM as the differences between median OS calculated for specific GPA index levels were not significant enough to allow clear discrimination between subgroups. Patrikidou's group developed a sarcoma-specific GPA based on the histology, number of brain lesions, and patient's performance status, allowing the identification of patients who are more likely to benefit from the direct treatment of BM (Table 2) (Patrikidou et al. 2020). The final index uses 4-point cut-offs for the prognostic group levels (scores 0–1, 1.5–2.0, 2.5–3 and 3.5–4), with the GPA1 group (score 0–1) having the worst prognosis and the GPA4 group (score 3.5–4) having the better prognosis.

**Table 2:** Sarcoma Graded Prognostic Assessment (Sarcoma-GPA) index

	Score			
	0	0.5	1	2
Histology	adipocytic tumours, including liposarcoma and myxoid liposarcoma	Smooth muscle tumours, skeletal muscle tumours, fibroblastic/myofibroblastic tumours, fibrohistiocytic tumours, vascular tumours, tumours of uncertain differentiation including intimal sarcoma	Tumours of uncertain differentiation, including synovial sarcoma, clear cell sarcoma, epithelioid sarcoma, small round cell tumours, undifferentiated sarcomas, and also malignant peripheral nerve sheath tumour/neurofibrosarcoma and phyllodes tumour/cystosarcoma of the breast	Alveolar soft part sarcomas and solitary fibrous tumours (SFT)/hemangiopericytoma
ECOG PS score	3–4	2	0–1	–
Number of CNS metastases	> 4	2–4	1	–

The modality of treatment affecting OS is difficult to evaluate due to insufficient numbers of patients undergoing specific therapies. Many researchers have agreed that the overall survival is more prolonged for patients undergoing surgery. After metastasectomy, the mean OS varies from 5.4 to 25.8 months (Chou et al. 2011; Espat et al. 2002; Salvati et al. 2010; Yoshida et al. 2000). Chemotherapy is usually implemented in the postoperative setting. Therefore, the effect of its sole use remains unclear. Two studies have identified a survival benefit with the use of chemotherapy (Al Sannaa et al. 2016; Chaigneau et al. 2018), while others have not (Gusho et al. 2021).

In Salvati et al. study, 31% of BM locally recurred within a median of 3.6 months. It was more likely to happen in the case of haemorrhagic foci than non-hemorrhagic (38% vs 27%, respectively). The time interval to local recurrence has also appeared to be shorter in the hemorrhagic lesions (Salvati et al. 2010). It was later confirmed by Al Saannaa et al.; however, according to their research, local recurrence did not affect survival (Al Sannaa et al. 2016).

An important factor affecting the OS seems to be the performance status assessed with KPS or ECOG scale (Bindal et al. 1994; Salvati et al. 2010) (Patrikidou et al. 2020). In Salvati's study, the OS for patients undergoing surgery with KPS > 60 was 12.8 months versus 5.4 months for those with a KPS < 60. On the contrary, according to Deguchi's research, neither pre- nor postoperative KPS was a significant prognostic factor for OS, even though metastasectomy vastly improved postoperative KPS in 50% of the patients (Deguchi et al. 2020).

The overall survival by sarcoma histology has been reported only for some subtypes, and most of these data come from small studies or case reports. The values are as follows: 3–18 months in leiomyosarcoma (Dietel et al. 2021), 1–18 months in angiosarcoma (Zakaria et al. 2015), a median of 4 months in osteosarcoma (Zhu et al. 2021), a mean of 32.3 months in ASPS (Gonzalez et al. 2021) and 2.7 months in Ewing sarcoma and rhabdomyosarcoma (Parasuraman et al. 1999).

## Conclusions

Brain metastases in sarcoma are rare, but some histological subtypes, including undifferentiated pleomorphic sarcoma, ASPS, leiomyosarcoma and osteosarcoma, have a higher propensity to metastasize to the brain. Brain metastases are usually preceded by metastases to other sites, most commonly the lungs.

The majority of patients report neurological symptoms before brain metastasis diagnosis, and it is usually based on neuroimaging. Although current guidelines do not recommend routine brain imaging for most sarcoma patients without neurological symptoms, CNS MRI or CT should be performed in patients with ASPS and could be considered for clear-cell sarcoma and angiosarcoma.

Treatment options include surgery, radiotherapy and chemotherapy, with many patients receiving multimodal therapies. Surgery is preferable in case of resectable metastases, with some evidence of improved overall survival and reduced neurological symptoms. Radiotherapy is widely used in the therapeutic and palliative treatment of sarcoma BM. Stereotactic radiosurgery has been shown to give good local control of brain lesions, while whole-brain radiotherapy can improve survival when combined with localized therapies. Poor blood–brain barrier penetration of chemotherapeutic agents used in sarcoma treatment limits their effectiveness in treating brain metastases. However, some studies found survival benefits of chemotherapy, especially combined with metastasectomy. Despite the advances in modern cancer treatment, the outcome for sarcoma patients with brain metastases remains poor, with most patients succumbing to the disease in a few months.

The rarity of brain metastases in sarcoma and the lack of standard treatment result in various treatment combinations, making evaluating the effect of individual treatment modalities difficult. There are few extensive studies of sarcoma patients with BM, with most data coming from case reports, further limiting the possibility of drawing conclusions regarding the survival benefit of each therapy. More studies are needed to establish optimal treatment options in different histological subtypes of sarcoma metastatic to the brain.
